# Diagnostic value of endoscopic appearance during ductoscopy in patients with pathological nipple discharge

**DOI:** 10.1186/s12885-017-3288-3

**Published:** 2017-05-02

**Authors:** Ye Han, Jianyi Li, Sijia Han, Shi Jia, Yang Zhang, Wenhai Zhang

**Affiliations:** 1 Mammary Surgery Department, Shengjing Hospital of China Medical University, Shenyang, Liaoning Province People’s Republic of China; 2 Department of Breast Surgery, Shengjing Hospital, China Medical University, Shenyang, 110004 China

**Keywords:** Breast cancer, Pathological nipple discharge, Ductoscopy

## Abstract

**Background:**

To explore the features of ductoscopic appearance that may be diagnostic in patients with pathologic nipple discharge (PND) and to discuss the diagnostic criteria for intraductal tumors.

**Methods:**

We reviewed 247 patients with PND but without a palpable mass who were evaluated using either surgical biopsy or excision. Data concerning patient age, duration of discharge, discharge color, and the details of endoscopic appearance were analyzed according to the pathological results.

**Results:**

The postoperative diagnosis in 61 patients (24.70%) was a nonmass lesion, and 186 patients (76.52%) had an intraductal tumor. Among those with intraductal lesions, 10 patients (4.05%) had a malignant tumor, including 4 (1.62%) with ductal carcinoma in situ and 6 (2.43%) with invasive ductal carcinoma. On univariate analysis, patients of older age with spontaneous and bloody discharge were more likely to suffer from intraductal lesions. On logistic regression analysis, bloody nipple discharge, morphology, and a broad lesion base revealed by ductoscopy showed a statistically significant correlation with malignancy (*p* = 0.001, *p* < 0.001, *p* = 0.022, respectively).

**Conclusions:**

Both clinical features and endoscopic appearance are significant for the precise diagnosis of an intraductal lesion seen on ductoscopy. The endoscopic features of bloody discharge, morphology, and a broad lesion base are independent risk factors for malignancy and represent new criteria for the diagnosis of patients with PND.

**Electronic supplementary material:**

The online version of this article (doi:10.1186/s12885-017-3288-3) contains supplementary material, which is available to authorized users.

## Background

Pathologic nipple discharge (PND) is defined as unilateral, nonphysiologic nipple discharge from a single duct unit. This symptom is reported in 5% to 8% of breast-clinic consultations [1, 2]. Papilloma, as the most common cause, accounts for between 40% to 70% of the etiology of PND, followed by adenomatous or papillary epithelial proliferation. Reportedly, 5% to 15% of women with PND are diagnosed with breast cancer or ductal carcinoma in situ (DCIS) [3, 4]. Mammary fiberoptic ductoscopy is used worldwide as a standard method of diagnosis for PND; however, there is no consensus on the utility of evaluating the endoscopic appearance. The aim of this study is to discuss the features of endoscopic appearance that are related to a tendency toward malignancy and to create a diagnostic model for PND using ductoscopy.

Mammary fiberoptic ductoscopy was first described in 1989 as an effective examination for diagnosing the cause of nipple discharge in women [5, 6]. The development of ductoscopy proceeded from directly inserting a scope into the nipple orifice with visualization of the mammary ductal epithelium, to eventual biopsy capabilities with cytological analysis of intraductal lesions. The initial rigid ductoscopes had a diameter of more than 1.5 mm, but rapidly developing technology has given us the opportunity to use fiberoptic ductoscopes with smaller diameters (0.55–1.1 mm) [7]. Many examination modalities are used to make a diagnosis in patients with PND: mammo*g*raphy (MG), ultrasonography (US), galactography, and nipple-discharge cytology. However, there are no definite criteria for diagnosing PND, and each examination has its limits.

We designed this study to examine the utility of the ductoscopic appearance in diagnosing PND. We examine the correlation of ductoscopic appearance with malignant features in order to predict the malignant inclination of a lesion. We also discuss the indications for surgery in patients with PND.

## Methods

This retrospective study included 247 patients (aged 23–76 years) who complained of PND. All patients were seen at our surgery clinic between July 2010 and September 2013 and underwent ductoscopy followed by open biopsy or target-duct excision. Informed consent for ductoscopy and biopsy was obtained from each patient. All patients were examined by breast US and MG before ductoscopy, and nonbreast causes of PND, such as hyperprolactinemia and inflammatory processes, were ruled out by laboratory evaluation. PND was divided into 4 groups by appearance: serous, whitish, bloody, and brown. The patients with PND or abnormal imaging results were given ductoscopy and following open biopsy under general anethesia. We used ductoscopes manufactured by Schölly Fiberoptic GMBH (Denzlingen, Germany). The endoscopes were 10 cm in length and had a diameter of 0.6–0.8 mm. The working channel could accommodate tools such as biopsy forceps. After ductoscopy, either the defined tumor was removed or the target duct was excised. When tumors were clearly seen on ductoscopy, we documented the location, depth from the orifice, quadrant, morphology, presence of hemorrhage, and the size of the lesion base.

### Operative technique

The nipple-areola complex was cleaned with a povidone iodine solution, and ductoscopy was performed under local anesthesia with diluted lidocaine (0.5%). First, a blunt pinhead with a diameter of 0.1 mm was placed into the dilated ductal orifice. An expander system was then introduced into the ductal orifice to gently expand the duct. Finally, the fiberoptic scope was introduced.

Selective ductectomy was performed on the basis of suspected intraductal disease. After local anesthesia was administered and blue dye injected through a 24-gauge cannula, an infra-areolar incision was made, and the areolar flap was raised. The pathologic duct was identified and the dyed portion, 3 or 4 cm in length, was removed with a small margin of surrounding breast tissue. The specimens were oriented with a short suture at the lesion site and a long suture at the end of the pathologic duct. All removed ducts were sent for histological examination.

### Histological analysis

The tissue was fixed using 4% buffered formalin and sliced from the central part to the periphery, then blocked in consecutive transsectional planes and stained with hematoxylin and eosin. The examination results were categorized as ductal hyperplasia, isolated papilloma, papillomatosis, papilloma associated with atypical ductal hyperplasia (ADH), ductal carcinoma in situ (DCIS), and papilloma associated with invasive ductal carcinoma (IDC).

### Data and statistical analysis

All data were collected retrospectively. MG and US results were classified using the Breast Imaging Reporting and Data System (BIRADS). Normal results were ranked as BIRADS 0–3, while abnormal results received BIRADS 4–5 distinction. Since all patients underwent either open biopsy or target-duct excision, we could determine how the histology of the intraductal lesion correlated with the visual description on ductoscopy. Data were analyzed using Statistical SPSS Version 16.0 (IBM, Chicago, JSA). The following variables were analyzed using chi-square analysis: patient age, duration of nipple discharge, location of discharge, color of discharge, and whether the discharge was spontaneous. Associations between intraductal papillary lesions and all potential variables were assessed using univariate analysis followed by multivariate analysis of the meaningful subsets. Logistic regression analysis was then used to explore the relation between the visual description of the lesion and the histopathology results. Associations between the predictors and the papillary lesions were quantified by calculating the odds ratios with 95% confidence intervals for each variable.

## Results

**Fig. 1:**
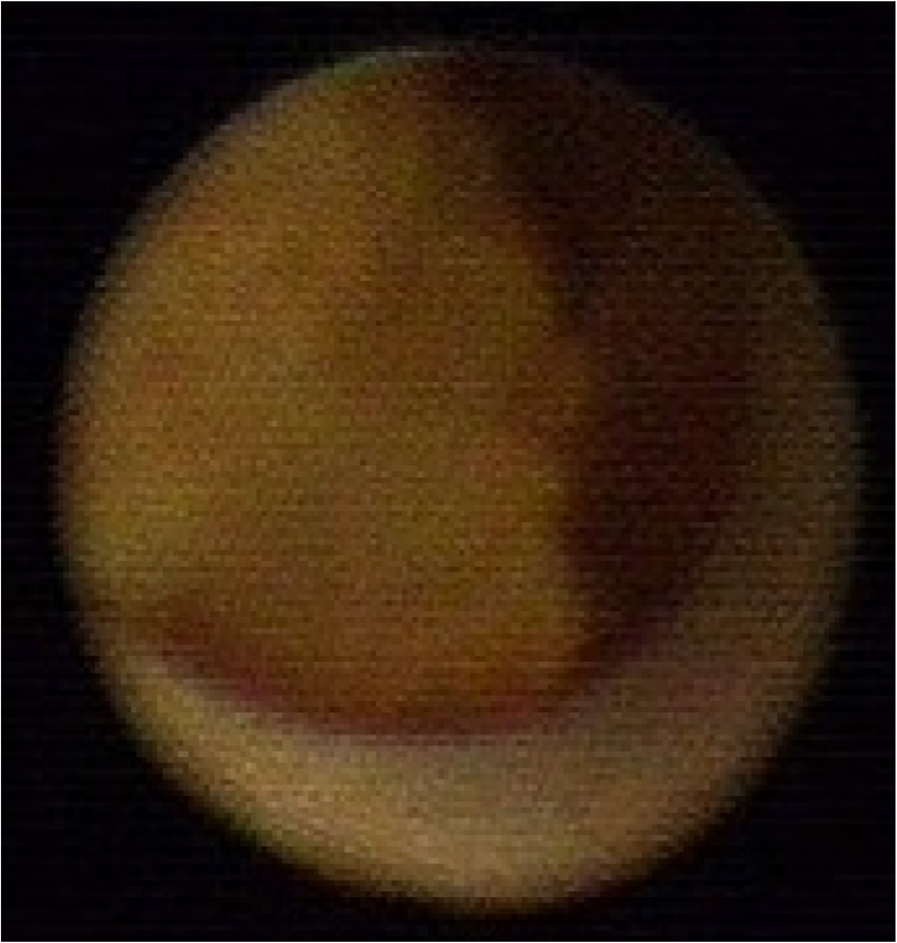
Ductoscopy image of a benign solitary papilloma. Legend: Irregular shape, no hemorrhage, narrow base

**Fig. 2:**
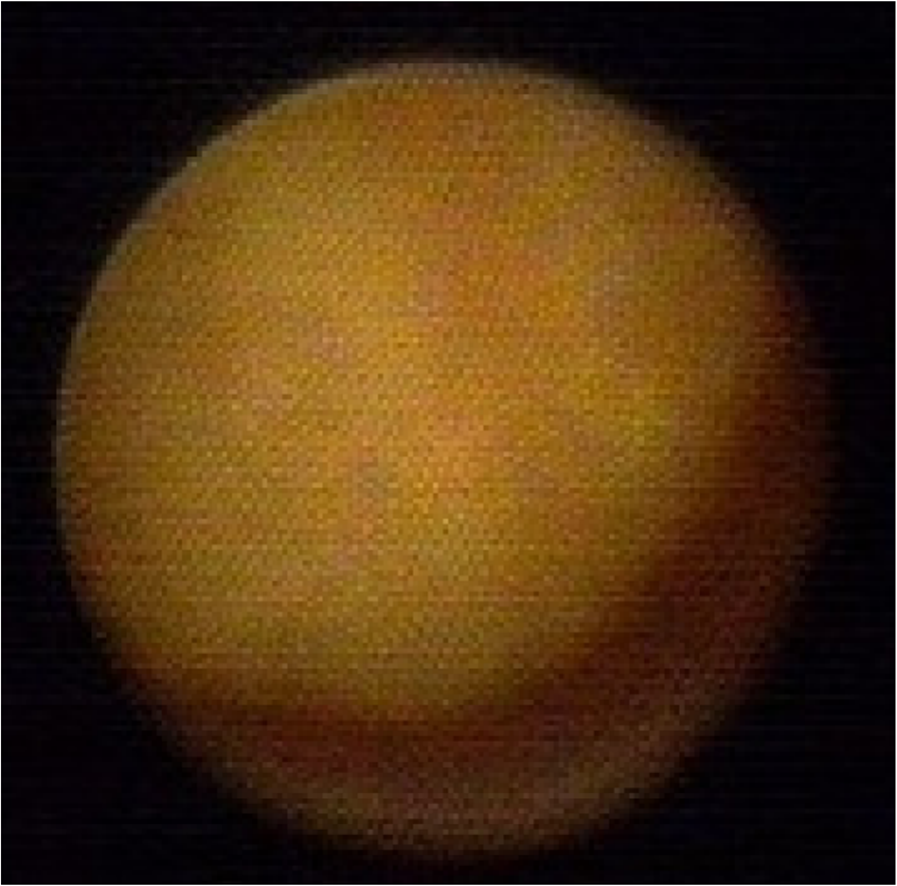
Ductoscopy image of a benign solitary papilloma. Legend: Round shape, no hemorrhage, narrow base

**Fig. 3:**
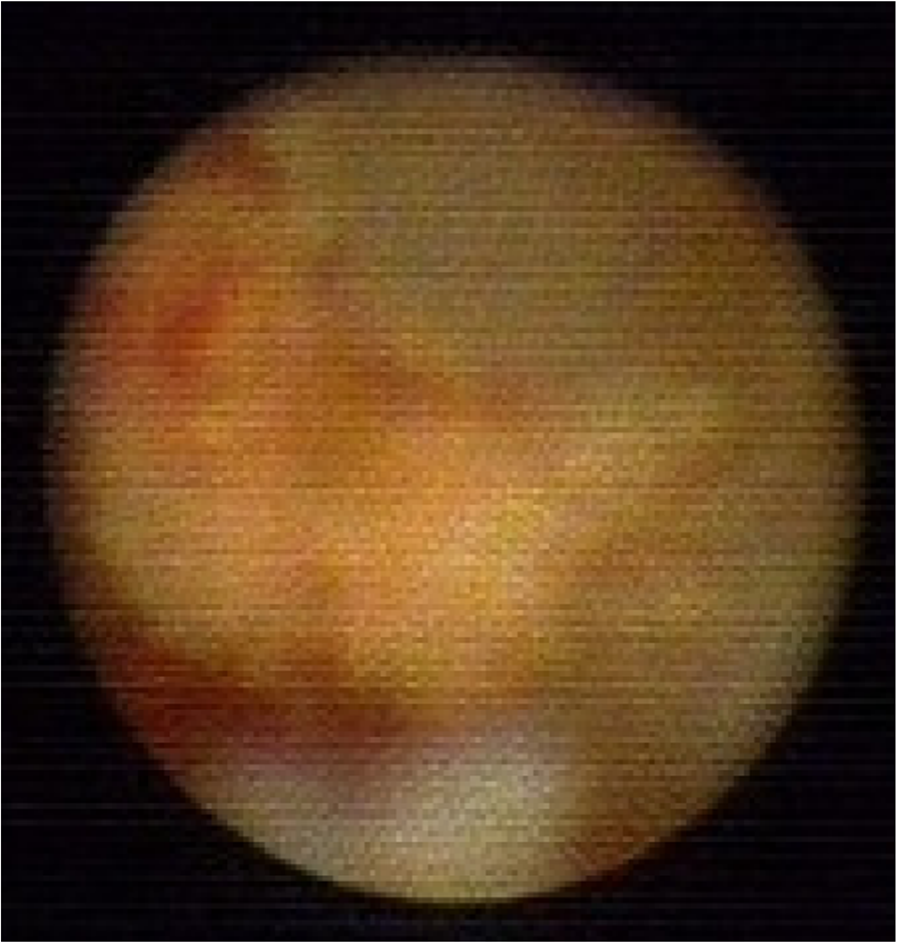
Ductoscopy image of a benign solitary papilloma. Legend: Irregular shape, no hemorrhage, narrow base

**Fig. 4:**
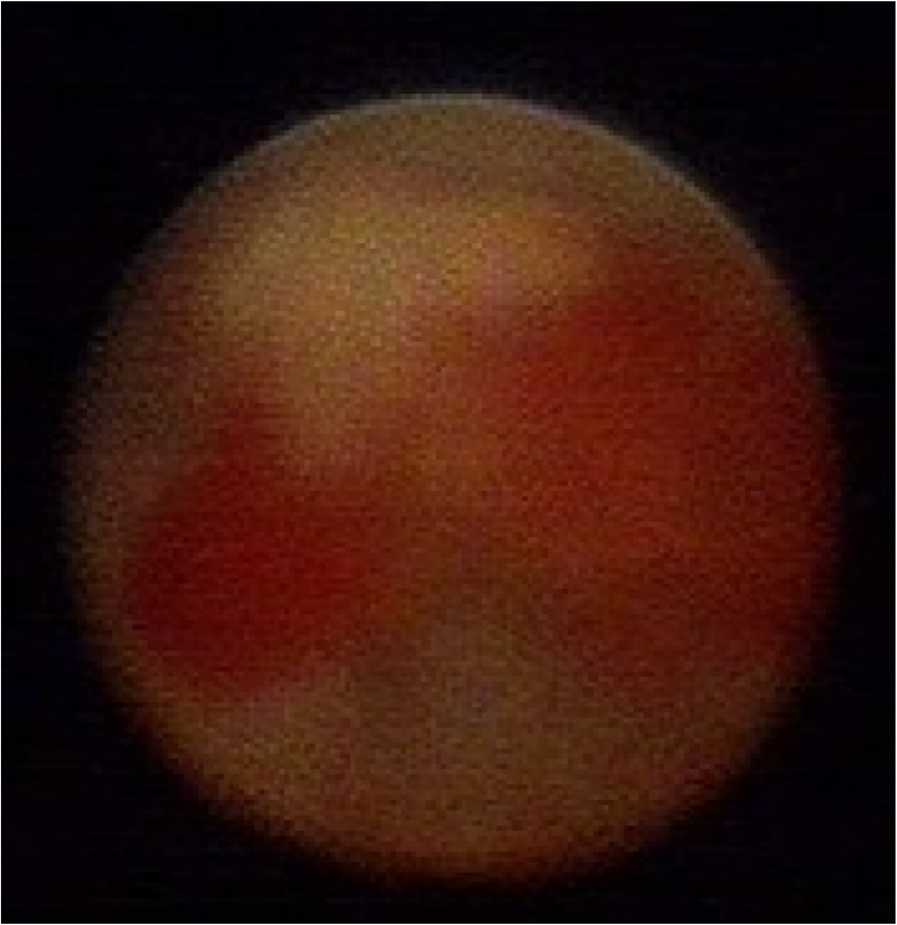
Ductoscopy image of a solitary papilloma with ADH. Legend: Irregular shape, hemorrhage, narrow base

**Fig. 5:**
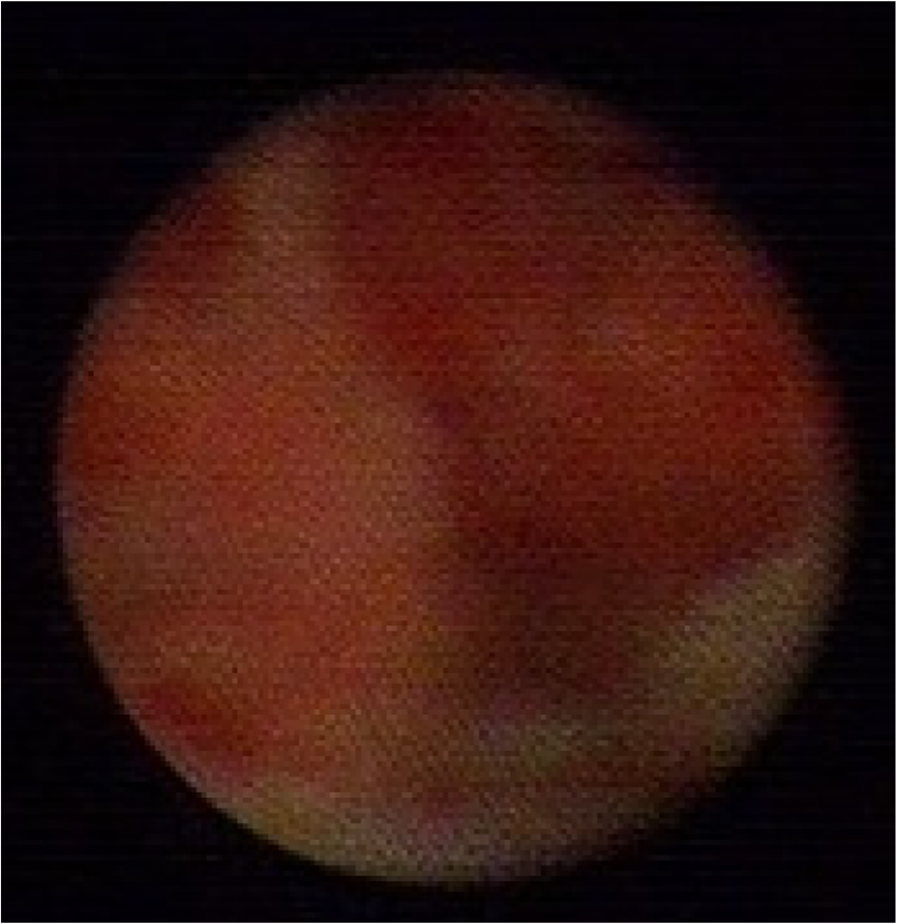
Ductoscopy image of a solitary papilloma with ADH. Legend: Irregular shape, hemorrhage, broad base

**Fig. 6:**
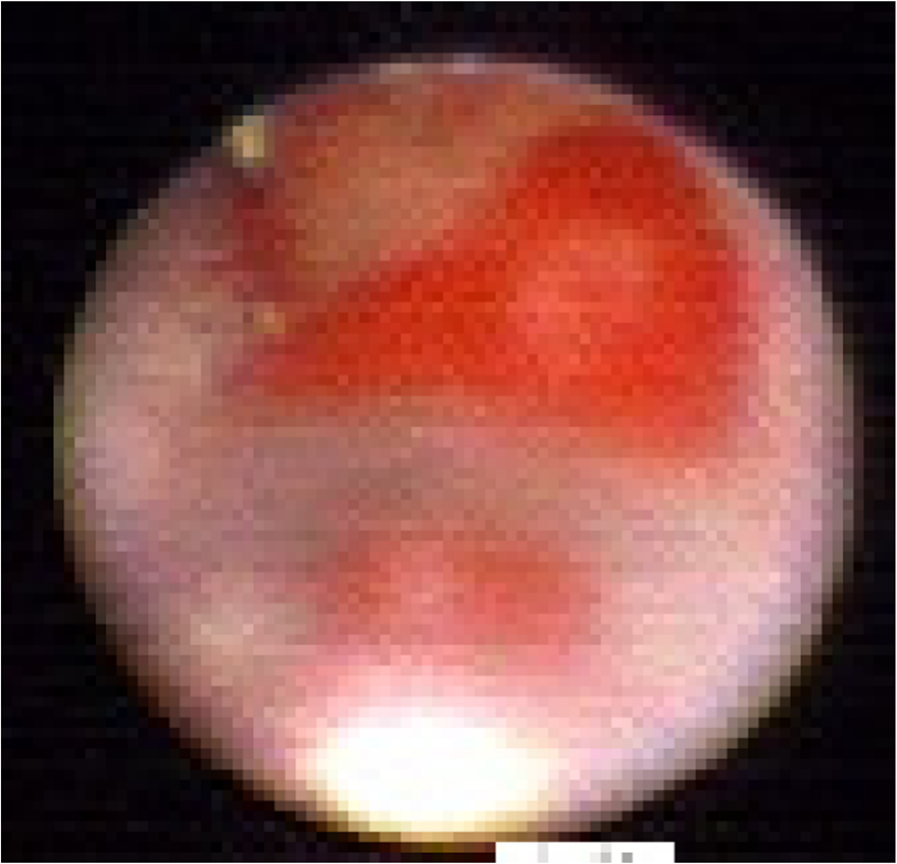
Ductoscopy image of a IDC. Legend: Strawberry like shape, hemorrhage, broad base

**Fig. 7:**
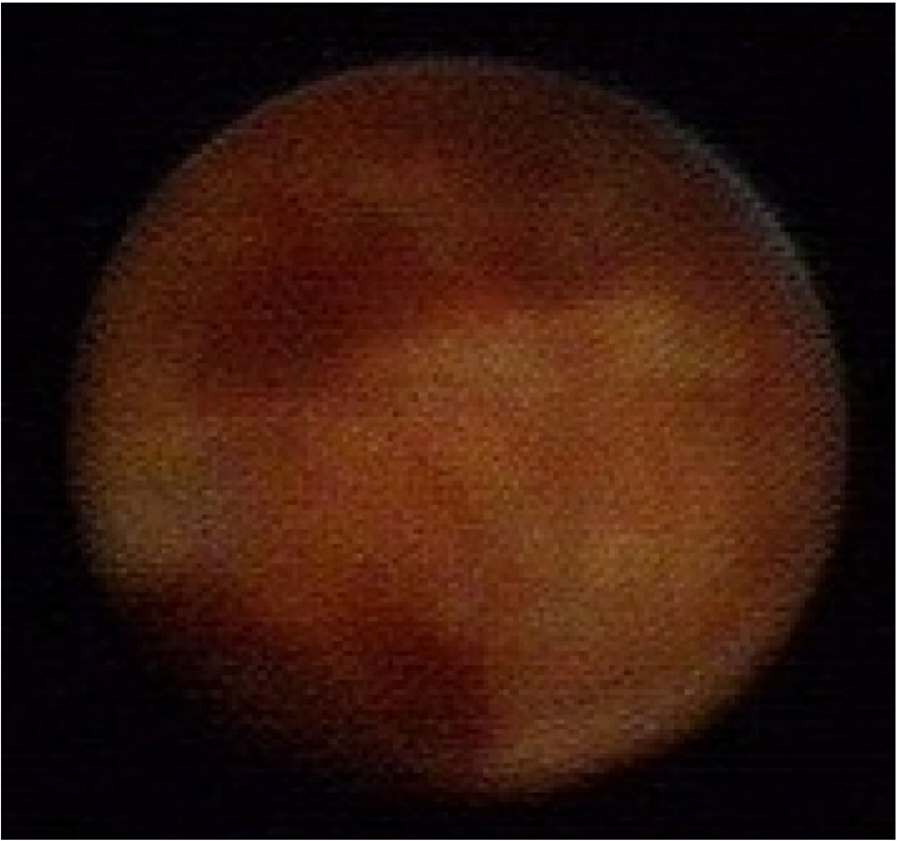
Ductoscopy image of a DCIS. Legend: Uneven with stiffness, no hemorrhage, broad base

**Fig. 8:**
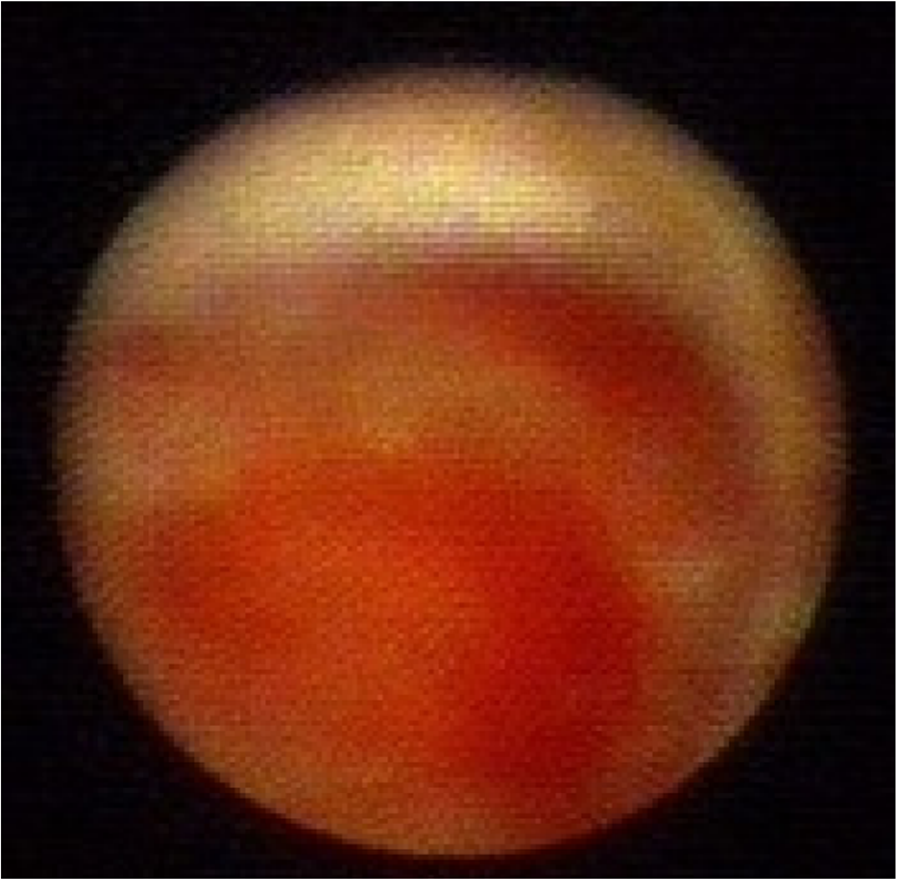
Ductoscopy image of a solitary papilloma with ADH. Legend: Irregular shape, hemorrhage, broad base

**Fig. 9:**
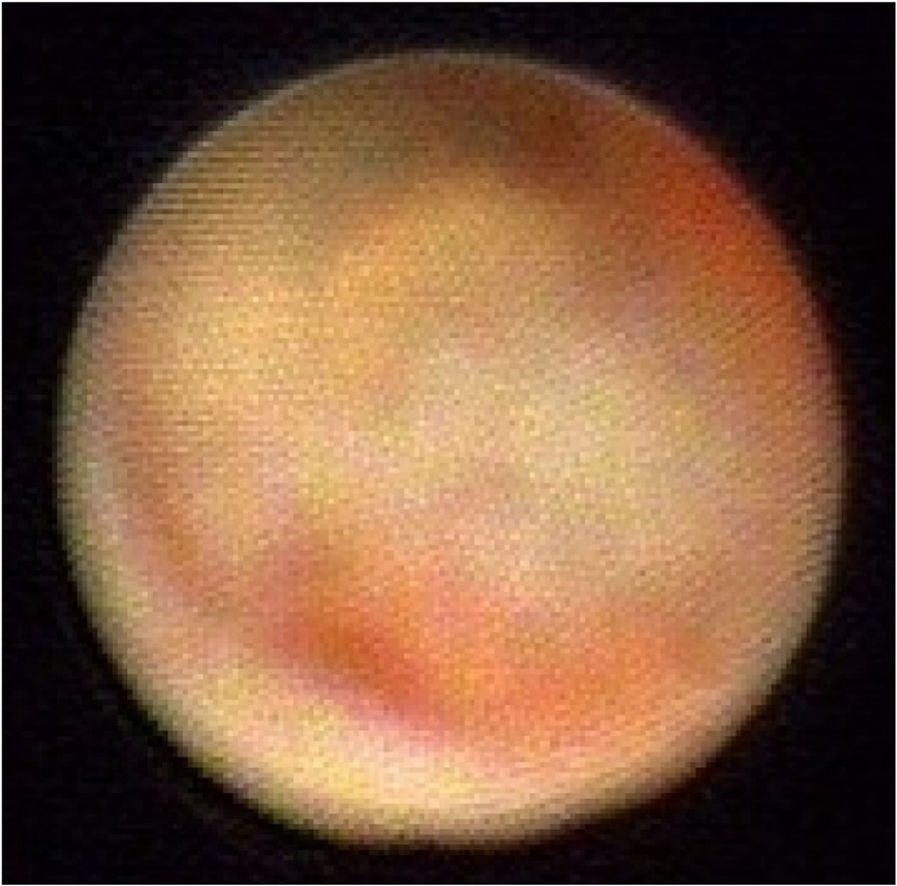
Ductoscopy image of a DCIS. Legend: Uneven with stiffness shape, hemorrhage, broad base

Ductoscopy was successfully performed in 247 patients with unilateral PND. Nine typical endoscopic pictures of ductoscopy findings are provided, with detailed descriptions (Figs. 1, 2, 3, 4, 5, 6, 7, 8 and 9). A total of 121 patients complained of left-sided PND, 126 complained of right-sided PND. The duration of solitary nipple discharge ranged from 1 month to 3 years, with a mean duration of 5.77 months. According to the patients’ descriptions, the color of the discharge was divided into a nonbloody group with 75 patients (30.36%) and a bloody group with 172 patients (69.64%).

In order to fully assess the role of ductoscopy as a standard diagnostic modality for unilateral PND, all patients were examined and evaluated using both US and MG. Of the 186 patients with intraductal lesions, mammographic abnormalities were present in 8.60% (16/186), and US abnormalities were present in 67.74% (126/186). Of the 61 patients with ductal ectasia, MG showed abnormalities in 52.46% (32/61), and US showed abnormalities in 31.15% (19/61). All intraductal lesions were removed, and we were able to obtain final pathologic results. For patients with unilateral PND the intraductal-tumor detection rate, based on the results of ductoscopy and final pathology, was 76.52%, and the malignant-tumor detection rate was 4.85%. Histopathologic investigation of the surgically excised lesions in the patients who complained of solitary nipple discharge revealed that 7 patients had papillomatosis, 95 had solitary papilloma, 74 had papilloma with ADH, 4 had DCIS, 6 had IDC, and 61 had ductal ectasia or galactophoritis (Table 1).

**Table 1:** Pathological diagnosis after surgery

Histopathology	N(247)	Ratio
Ductal hyperplasia	61	24.70%
Intraductal papilloma	95	38.46%
Papillomatosis	7	2.83%
Papilloma + ADH	74	29.96%
DCIS	4	1.62%
Invasive carcinoma	6	2.43%

Detailed tumor data were analyzed by logistic analysis using the location of the tumor, depth of the lesion, distance between the orifice and the tumor, quadrant of the tumor, morphology, the presence of blood in the discharge, and the size of the tumor base. Our study demonstrated that patients with bloody nipple discharge were more likely to have intraductal papillary lesions than those with nonbloody nipple discharge (Table 2; *p* < 0.01). Patient age and spontaneity of the discharge were independent factors related to malignancy (0.01 < *p* < 0.05). Using Spearman’s test, the endoscopic features of location, lesion depth, quadrant, and hemorrhage were not related to the malignancy of the tumor (Table 3; *p* = 0.273, *p* = 0.309, *p* = 0.981, *p* = 0.544, respectively). The morphology and a broad tumor base were correlated with malignancy (Table 3; *p* < 0.001, *p* < 0.001, respectively). Bloody nipple discharge, morphology, and a broad tumor base showed statistically significant correlations with the malignant tendency of the tumor (Table 4; *p* = 0.001, *p* < 0.001, *p* = 0.022, respectively).

**Table 2:** Univariate analysis of factors associated with intraductal papillary lesion identified by ductoscopy by chi-square test

Factors	Diagnosis made by pathology		*X* ^2^	*P* value
	Papillary lesion	Nonpapillary lesion		
Age (year)				
< 40	59	28	4.05	0.033
≥ 40	127	33		
Duration of discharge (month)				
≤ 6	127	38	0.74	0.239
> 6	59	23		
Color of discharge				
Non-bloody	36	39	43.17	*P* < 0.01
Bloody	150	22		
Spontaneous or not				
Spontaneous	181	54	5.89	0.011
Non-spontaneous	5	7

**Table 3:** Spearman test of factors associated with benign and malignant intraductal papillary lesion identified by ductoscopy according to pathological results

		Diagnosis by pathology				*p*	*r*
		SP	MP	DCIS	IDC		
Location	1st branch	81	1	1	4	0.273	−0.081
	2nd branch	60	3	1	1		
	3rd branch	17	2	1	2		
	4th branch	11	1	0	0		
Depth	<=2 cm	65	0	1	3	0.309	−0.075
	>2 cm	106	7	3	3		
Quadrant	upper outer	45	3	1	4	0.981	0.002
	lower outer	34	3	0	2		
	upper inner	40	1	1	0		
	Lower inner	38	0	1	0		
	Behind nipple	14	0	1	0		
Morphology	round	43	2	0	1	0.000	0.523
	irregular	75	4	2	3		
	strawberry	47	0	1	2		
	Uneven stiffness	4	0	1	0		
Hemorrhage	Less or no	160	6	3	4	0.544	0.045
	more	11	1	1	2		
Lesion base	narrow	110	4	1	1	0.000	0.294
	broad	61	3	3	5

**Table 4:** Multivariate analysis of factors associated with intraductal papillary lesions identified by ductoscopy by logistic regression

	*P* value	95% *CI*
Color of discharge	0.001	0.379-1.444
Morphology	0.000	0.782-1.517
Lesion base	0.022	0.117-1.475

### Characteristics of patients with cancer

A total of 10 patients (4.05%) with intraductal tumors were found to have malignant disease. The features of these patients are shown in Table 5. All patients with malignancy had spontaneous bloody nipple discharge and lower human epidermal growth factor receptor-2 (HER-2) expression. Six patients had irregularly shaped lesions. One lesion was strawberry-like, and 3 lesions had an uneven shape with stiffness typical of malignancy. About 80% of patients with broad-based lesions had tumors with malignant features. Seven patients had high estrogen-receptor (ER) or progesterone-receptor (PR) expression, although this was not significantly different between groups. The diameter of the IDC lesions was larger than that of the DCIS lesions (*p* < 0.05).

**Table 5:** Ductoscopic Appearance of malignant lesions

		Characteristics of malignant tumors		*P* value
		DCIS	IDC	
Age	≦40 years old	1	1	0.667
	> 40 years old	3	5	
Spontaneous	Spontaneous	4	5	0.600
	Non-spontaneous	0	1	
Bloody	Bloody	4	6	0.317
	Non-bloody	0	0	
depth	≦3 cm	4	5	0.600
	>3 cm	0	1	
Quadrant	upper outer	1	3	1.000
	upper inner	1	0	
	Lower outer	0	2	
	Lower inner	1	0	
	Behind nipple	1	1	
Morphology	round	0	1	1.000
	irregular	2	3	
	strawberry	1	2	
	stiffness	1	0	
Hemorrhage	Less or no	3	4	0.667
	more	1	2	
Lesion base	narrow	1	1	0.667
	broad	3	5	
Diameter	≦1.0 cm	4	1	0.024
	>1.0 cm	0	5	
ER	+/++	3	4	0.667
	-	1	2	
PR	+/++	3	4	0.667
	-	1	2	
HER-2	+	0	1	0.600
	-	4	5	

## Discussion

Our study shows that ductoscopy is the most effective examination for PND, with relatively high sensitivity and specificity. PND can be associated with early breast cancer, and diagnosis by fiberoptic ductoscopy is recommended worldwide [8]. Sarakbi reported that approximately 50% of patients with a breast papilloma have PND, and 5% to 17% of papillomas will eventually turn malignant [9]. PND is the main complaint in 0.5% to 12% of malignant breast lesions, especially DCIS and IDC [10]. One study demonstrates that approximately 80% to 85% of breast cancers originate from the epithelium of the mammary ducts, with IDC developing from the initial stage of an intraductal tumor; the authors call for new methods of diagnosis based on mammary duct involvement [11]. Conventional examination employs indirect methods such as MG, breast US, and magnetic resonance imaging (MRI), but MG and US are no longer recommended examination modalities for intraductal lesions that cause PND [10, 12]. In our study, the sensitivity and specificity of MG are 8.6% and 95%, and for US they are 67.74% and 68.85%. All available data support the conclusion that indirect examination is not specific or effective for PND. However, ductoscopy is an emerging examination modality that is capable of direct visualization of the source of the PND; it deserves detailed discussion and further attention. Fiberoptic ductoscopy is recommended worldwide as a direct and effective diagnostic test [12], and its application is gradually being promoted in the clinic.

In this retrospective study, we successfully examined 247 patients with single-duct PND from our department and found that the incidence of intraductal lesions was 75.30% (186/247), which is consistent with the detection rate of 77% reported by Khan [13]. Liu et al. showed that intraductal lesions are found on ductoscopy in 63.2% of patients with PND [14]. Intraductal papillomas are benign breast lesions covered by the epithelial and myoepithelial cell layers; they account for 1% to 2% of breast neoplasias and 10% of benign tumors [15, 16]. Most intraductal papillomas are small, less than 5 mm in diameter; our study detected a mean diameter of 0.69 mm. The majority of solitary papillomas are benign (71.66%), although they can be associated with cytological atypia (29.55%), DCIS (2.02%), or invasive malignancy (2.83%). PND is the primary symptom of a intraductal tumor, with bloody discharge most commonly seen (69.64%), while serous discharge ranks second (24.29%). Most discharge results from central lesions (81.62%), rather than peripheral disease. Solitary intraductal papillomas, the main cause of PND, are usually located within the central breast tissue in patients of middle age (40–50 years), whereas papillomatosis is frequently found in the peripheral tissue and in younger patients [17].

Mammary ductoscopy is an evolving technology. It enables direct observation of the duct cavities and duct walls, but detailed criteria for description of its findings are not yet defined. Although the 2002 Japanese guideline broadly divides intraductal lesions into polypoid-solitary type, polypoid-multiple type, combined type, and superficial type [18, 19], there is no universal consensus on any particular endoscopic appearance that is associated with malignancy. Al Sarakbi et al. [20] graded lesions (D0–D5) based on the degree of suspicion for malignancy of a given lesion. A grading system devised by Makita does not precisely correspond with histologic diagnosis, while Al Sarakbi’s system requires the expertise of a surgeon rather than an objective description [14]. Our study specifies the detailed objective ductoscopy features that are closely related to malignancy of a lesion. We divide the morphology into 4 types: round, irregular, strawberry-like, and uneven with stiffness. The base of the lesion is divided into narrow and broad groups according to whether the diameter of the base is larger or smaller than that of the lesion. These findings are able to be directly visualized during ductoscopy, but our findings will require further study before their importance is verified.

We found that bloody discharge, spontaneous discharge, lesion morphology, and a broad tumor base had a significant connection with the diagnosis. Continual bloody discharge usually results from the extended capillaries around the tumor or from the infiltrating of normal tissue by malignant cells. The shape of a lesion always yields a great amount of information. For example, a round, regular shape is inclined to be a benign feature, while a strawberry-like and uneven, stiff lesion covered by hyperplastic epithelium cells or sharp spikes of tissue is highly likely to be malignant (*p* < 0.001). Our findings regarding the strawberry shape and lesion stiffness are similar to those of Makita et al. [18]. They recognized that localized and sharp-edged polypoid lesions tend to be benign, while lesions with multiple areas of flat elevations and a surrounding rough surface are always breast cancer. However, their theory of shape discusses just one aspect of a lesion, so its clinical significance is limited. We found that a broad tumor base is a significant factor, indicating a diagnosis of malignancy (0.01 < *p* < 0.05). The significant feature of epithelial-tumor proliferation is that tumor cells accumulate and multiply in physical size and number under the control of intracellular signaling networks, favoring a hypoxic microenvironment [21]. This theory explains the increasing incidence of malignancy with increasing tumor-base size. Makita argued that the polypoid-multiple type lesions should always be biopsied [18, 22] and that almost all lesions with a combined or superficial type on mammary ductoscopy are malignant. The elevated lesions were formed by intraductal cancer spreading continuously along the mammary ducts. We propose that a broad lesion base is a statistically independent factor that distinguishes a malignant focus from the benign majority.

There is a wide divergence of opinion on the treatment of intraductal lesions. We perform a wide, wedge-shaped incision, removing the offending papilloma including the main duct and surrounding tissue. Atkins and Wolff purport that papilloma removal is adequate and have developed the technique of microdochectomy, removing a single duct through a small incision following the circular line of the areola [23]. Some studies concentrate on ductoscopic papillomectomy and intraductal biopsy for benign tumors. Faith studied 26 patients with histopathologically confirmed papillomas and removed 22 endoscopically [24]. However, Kamali recognized the possibility of retained papilloma tissue using this modality. Kamali’s study found that the disease is usually underestimated, and disease progression is not hindered, by ductoscopic biopsy [14]. Researchers showed that the incidence of cancer evolving from the location of the pathological duct in the polypoid-solitary, multiple-lesion, superficial, no-lesion, and unclassified types is 8.5%, 19.0%, 31.6%, 7.2%, and 21.9%, respectively [25]. Atypical papillomas coexist with carcinoma in 22% to 67% of cases; therefore, ductal papillomectomy is inadequate treatment for intraductal papillomas with ADH [26]. Liu et al. state that all intraductal lesions found by ductoscopy should undergo duct resection [14]. Both ductal papillomectomy and microdochectomy have limitations that require further discussion. As there remains the possibility of retained tissue evolving into malignancy and the risk of undiscovered lesions, we have a policy of lesion removal, including the involved main duct and surrounding tissue, in order to prevent the development of atypical hyperplasia [25, 27].

In this study, 10 malignant tumors were found, with an incidence of malignancy 4.05% (10/247) [Additional file 1]. A multicenter German study of 214 patients found DCIS in 10 patients (4.7%) and IDC in 1 patient (0.05%) [10], similar to our rate of 4.05%. In our study, the patient age ranged from 37 to 66 years, with an average age of 47.9 years. The average tumor diameter was 1.15 cm. Between DCIS and IDC, the diameter of tumor lesions was the only feature to show a significant variation (*p* < 0.05), which supports the theory of an underlying process of hyperplasia. In patients with DCIS, 50% show abnormalities on MG, US, and MRI. In patients with IDC, 66.7% show abnormalities of MG and US, and 50% have abnormalities on MRI. These data show the significance of ductoscopy as an early detection method for breast cancer. When intraductal lesions are small, it is difficult to perform biopsy. With surgery, the intraductal lesion is removed and malignancy may be proven pathologically. Subsequent lumpectomy is performed, sometimes with sentinel node biopsy. Only 2 of our patients had lymph node metastasis. The malignancies had some features in common, such as spontaneous discharge (with 1 exception), the location of the main duct, bloody discharge, and an irregular shape. Moreover, the pathological results showed that malignant tumors were more inclined to have a high ER to PR ratio (70%) and low HER2 expression. Some researchers have confirmed similar results to ours. Masujiro found, in 67 patients, that positive findings for ER and PR status were noted in 94% and 89.6%, respectively, and in a separate study of 40 patients, none had a HER-2 positive result [25, 28]. Daigo studied 25 patients with breast cancer via endoscopic ductoscopy and found a high ER to PR expression ratio (80%); however, the disease-free survival was not affected by the ER-to-PR status or the endoscopic appearance [29]. Katrina found that an average of 40% of DCIS lesions are HER2-positive, a finding that has therapeutic implications in the era of targeted therapy with trastuzumab [30]. Based on the results above, we recognized that malignant tumors that develop from the duct epithelium mostly express high levels of ER and PR, with low levels of HER2 expression. Therefore, we consider that the cancers evolving from a location related to the affected duct tend to be nonaggressive or slow growing. Whether benign intraductal lesions have a high expression of ER and PR still needs further investigation. In our study, 80% of patients were diagnosed with early-stage breast cancer and given endocrine therapy. The other 20% with lymph node metastasis were given chemotherapy, radiotherapy, and endocrine therapy. The patients with malignancy were followed from 12 months to 36 months after surgery, with no evidence of recurrence.

According to our results, the shape of a tumor is not an exclusive factor with which one is able to make a diagnosis, since irregular shapes, polypoid shapes, and uneven shapes with stiffness exist in various types of lesions. Masujiro recommended that multiple- or superficial-type lesions or atypical papillary lesions diagnosed using intraductal breast biopsy must be followed up carefully due to the association with malignancy [25, 31]. Several studies have suggested that, for intraductal neoplasms, the location of the lesion, an irregular appearance, a rough surface, or multiple lesions might be characteristic indicators of malignancy [32]. In our study, the ductoscopy features of irregular shape, a broad lesion base, and bloody discharge were independent risk factors for malignancy, findings that are similar to but more precise than those of these earlier studies. The diagnosis of malignancy has to be made using various features of the ductoscopy description, never just according to shape [33]. According to the Japanese classification system, superficial lesions accompanied by continuous luminal irregularity, hemorrhage, or an erosive surface are easy to recognize, but it can be difficult to detect such lesions in cases where the vessels are hidden beneath the luminal surface [34, 35]. That theory may explain the reason why 1 patient with a malignant tumor had a round lesion, little hemorrhage, and a narrow tumor base, findings that are typically descriptors of a benign tumor. What we see on ductoscopy is not the whole story, and our findings still need further validation.

## Conclusion

Ductoscopy plays an important role in the diagnosis of intraductal lesions. Its advantages include direct visualization of lesions and its high sensitivity and specificity [36]. Clinicians are in great need of a precise evaluation system that uses endoscopic appearance to improve the diagnostic ability of ductoscopy. Our study specifies the details of endoscopic appearance that are associated with malignancy: color of discharge, morphology, and lesion-base size are independent risk factors for malignancy. Malignant tumors found by ductoscopy are inclined to be DCIS rather than IDC and have higher ER and PR expression, which indicates that ductoscopy is an efficient method to discover breast cancer in its early stages. We note that all patients with malignancy had spontaneous bloody nipple discharge. We boldly assume, therefore, that patients with nonbloody nipple discharge may be safely followed. However, with hyperplastic change in the ductal epithelium, an intraductal lesion may progress to malignancy. Our study shows that 29.96% of patients have ADH, which has the inclination toward malignant transformation. Therefore, benign intraductal lesions should be closely followed and removed before pathologic change can occur. Even for some intraductal lesions with benign signs, we choose to make a wide-wedge incision since the possibility of atypical tissue exists. The insidious signs and the small size of intraductal lesions often impedes us from diagnosing and treating patients with PND, but we have determined that some signs deserve attention: severe bloody nipple discharge; irregular, strawberry-like, or stiff endoscopic appearance; and a broad lesion base. These findings demonstrate a high tendency toward malignancy. PND is commonly reported in clinics, but its significance as an early sign of intraductal disease or even malignancy may be neglected. Further investigation is still needed in order to establish a diagnostic system using ductoscopic appearance.

## Additional file

Additional file 1: Detail data of malignant tumors. Description of form: It is the detail data of malignant tumors, which includes age range, duration, spontaneous, quadrants, depth, endoscopic description, pathologic description and so on. They are the base data for analyzation. (XLS 19 kb)

## Abbreviations

ADH: atypical ductal hyperplasia
DCIS: ductal carcinoma in situ
ER: estrogen receptor
HER-2: human epidermal growth factor receptor-2
IDC: invasive ductal carcinoma
MG: mammography
MP: multiple intraductal papillomas
MRI: magnetic resonance imaging
PND: pathological nipple discharge
PR: progestin receptor
SP: solitary intraductal papilloma
US: ultrasonography
